# Leukocyte-Rich Platelet-Rich Plasma (L-PRP) Promotes Rejuvenation of Senescent Fibroblasts and Dermal Remodeling via CCL1-CCR8 Signaling and PKM2 Modulation

**DOI:** 10.3390/ijms27146281

**Published:** 2026-07-15

**Authors:** Seyeon Oh, Hyoung Moon Kim, Wook Oh, Gwahn Woo Cheon, Kyoungmi Lee, Kuk Hui Son, Kyunghee Byun

**Affiliations:** 1 Functional Cellular Networks Laboratory, Lee Gil Ya Cancer and Diabetes Institute, Gachon University, Incheon 21999, Republic of Koreamd.mac12@gmail.com (H.M.K.);; 2 LIBON Inc., Incheon 22006, Republic of Korea; 3 Maylin Apgujeong Clinic, Seoul 06024, Republic of Korea; 4 Department of Anatomy & Cell Biology, Gachon University College of Medicine, Incheon 21936, Republic of Korea; 5 Maylin Clinic the Hyundai, Seoul 07335, Republic of Korea; 6 Maylin Clinic, Ilsan, Goyang 10391, Republic of Korea; 7 Regain Clinic, Incheon 22002, Republic of Korea; 8 Department of Thoracic and Cardiovascular Surgery, Gachon University Gil Medical Center, College of Medicine, Gachon University, Incheon 21565, Republic of Korea; 9 Department of Health Sciences and Technology, Gachon Advanced Institute for Health & Sciences and Technology (GAIHST), Gachon University, Incheon 21999, Republic of Korea

**Keywords:** leukocyte-rich platelet-rich plasma, skin aging, CCL1/CCR8/PKM2 signaling, extracellular matrix regeneration, collagen synthesis

## Abstract

Platelet-rich plasma (PRP) is widely utilized for skin rejuvenation and tissue regeneration; however, its biological effects vary according to leukocyte content and molecular composition. We investigated the mechanism by which leukocyte-rich PRP (L-PRP) enhances extracellular matrix (ECM) regeneration in aged skin, focusing on the CCL1-CCR8/pyruvate kinase M2 (PKM2) signaling axis. First, we demonstrated that L-PRP contains significantly higher levels of CCL1 than platelet-poor plasma (PPP). In senescent human dermal fibroblasts, L-PRP increased CCL1-CCR8 interactions in a manner linked to enhanced formation and nuclear translocation of PKM2 dimers. This enhancement was accompanied by Janus kinase (JAK)/signal transducer and activator of transcription 3 (STAT3) pathway activation and the upregulation of STAT3-dependent anti-apoptotic proteins (Bcl-2, Bcl-xL) and proliferative markers (Cyclin D1), resulting in increased fibroblast proliferation. Furthermore, L-PRP increased PKM2 tetramer levels, promoted PKM2-SMAD7 binding, and reduced SMAD7-mediated inhibition of transforming growth factor (TGF)-β signaling, leading to enhanced SMAD2/3 activation. These molecular events augmented the synthesis of collagen types I and III in senescent fibroblasts. In aged mice, intradermal L-PRP injections elicited dose-dependent increases in fibroblast proliferation, collagen fiber deposition, and skin elasticity. Nuclear PKM2 dimer/STAT3 signaling and PKM2 tetramer/TGF-β signaling were both more strongly activated in the L-PRP group. Our findings identify PKM2 as a central metabolic and signaling integrator linking immune-derived chemokines to fibroblast rejuvenation. This study provides mechanistic insights concerning how L-PRP promotes ECM regeneration in aged skin via coordinated regulation of fibroblast survival, proliferation, and collagen synthesis.

## 1. Introduction

Skin aging is broadly categorized into intrinsic aging, which occurs naturally with advancing age, and extrinsic aging, primarily driven by chronic environmental factors such as ultraviolet radiation [1]. Despite these etiological differences, both types share a common clinical hallmark: progressive wrinkle formation and elasticity loss due to dermal atrophy [2]. A fundamental mechanism underlying this dermal atrophy is a substantial reduction in the extracellular matrix (ECM), particularly collagen types I and III [3]. In aged skin, ECM homeostasis is disrupted because collagen synthesis substantially declines while enzymatic degradation increases [4,5,6]. This net loss of collagen integrity is directly attributed to functional impairment of dermal fibroblasts, the primary cells responsible for ECM production. Crucially, senescent fibroblasts undergo morphological changes, including reduced cell size and spreading, which are closely associated with diminished biosynthetic capacity [7]. These structural alterations increase mitochondrial reactive oxygen species production [8]; they also lead to mechanical downregulation of the transforming growth factor (TGF)-β type II receptor [9]. Consequently, TGF-β signaling axis impairment serves as a central mediator of reduced ECM production, ultimately leading to structural deterioration of the dermis and the clinical manifestation of skin wrinkles [10].

Platelet-rich plasma (PRP) has been widely adopted in regenerative dermatology to enhance tissue repair by delivering a concentrated mixture of platelet-derived mediators and other blood-borne soluble factors [11]. Randomized clinical trial evidence indicates that intradermal PRP injections improve clinical parameters of photoaged facial skin relative to control injections [12]. PRP has also been used as an adjunct to device-based procedures (e.g., microneedling) for dermal remodeling, such as the approach utilized in atrophic acne scars, where meta-analytic data suggests additional benefit compared with microneedling alone [13]. Despite these applications, outcomes remain heterogeneous across studies, reflecting variability in PRP preparation methods and cellular composition [14].

A major source of PRP heterogeneity is leukocyte content. Leukocyte- and platelet-rich plasma (L-PRP) is defined by the presence of concentrated platelets and a substantial leukocyte fraction compared with leukocyte-poor preparations and platelet-poor plasma (PPP) [14]. Leukocyte-rich PRP preparations exhibit higher levels of inflammatory cytokines than pure PRP in comparative analyses, indicating that leukocyte inclusion can meaningfully alter the bioactive milieu delivered to tissue [15].

Accordingly, the biological effects of L-PRP may qualitatively differ from those of leukocyte-poor PRP, particularly in tissues where inflammatory chemokines couple immune signals to stromal cells, such as fibroblast activation [16]. Chemokine ligand/receptor signaling is increasingly recognized as a mechanism by which immune-derived cues directly regulate stromal cells. CCR8 is a CC chemokine receptor with CCL1 as a well-established ligand [17].

CCL1-CCR8 signaling has been implicated in tissue remodeling and fibrotic programs through macrophage recruitment and polarization, as well as direct effects on mesenchymal effector cells, with downstream engagement of pathways including JAK/STAT [18,19]. CCL1-CCR8 signaling has also been shown to induce hepatic stellate cell activation via the JAK/STAT pathway, promoting liver fibrosis [18]. Lung fibroblast proliferation and activation are driven in part by macrophage-derived mediators, including interleukin-10, CCL1, and TGF-β [20,21,22]. This interaction leads to excessive ECM production, accelerating the clinical progression of pulmonary fibrosis [23,24].

Recent evidence indicates that CCL1 can directly act on fibroblasts through CCR8, promoting anti-apoptotic signaling and metabolic reprogramming via pyruvate kinase M2 (PKM2), thereby influencing fibroblast survival and matrix production [25]. PKM2 is unique among glycolytic enzymes in that it exists in multiple oligomeric states—primarily tetrameric and dimeric/monomeric forms—each conferring distinct biological functions [26]. The tetrameric form exhibits high pyruvate kinase activity and primarily supports glycolytic flux and adenosine triphosphate production in the cytoplasm, favoring energy metabolism over transcriptional regulation [26,27].

In contrast, the dimeric or low-activity form of PKM2 displays reduced catalytic activity but acquires non-metabolic signaling functions, including nuclear translocation and interaction with transcriptional regulators [26,27]. Nuclear PKM2 can function as a protein kinase or transcriptional co-regulator, phosphorylating or binding to factors such as STAT3, β-catenin, and hypoxia-inducible factor-1α, thus promoting anti-apoptotic gene expression and cell cycle progression [28].

Recently, the PKM2 tetramer has been implicated in TGF-β signaling through a direct interaction with SMAD7, whereby PKM2 binding interferes with SMAD7-mediated negative regulation of TGF-β receptor signaling [29].

Based on the above findings, PKM2 appears to play a multifaceted role in fibrosis through its distinct oligomeric states. Specifically, the dimeric form of PKM2 may drive fibrotic progression by translocating to the nucleus and activating the STAT3 signaling pathway, which promotes cell survival and proliferation. Concurrently, the tetrameric form of PKM2 may exacerbate fibrosis by interacting with SMAD7, thus relieving TGF-β signaling inhibition and enhancing ECM synthesis.

L-PRP is characterized by the presence of monocytes, lymphocytes, and neutrophils, which results in higher levels of inflammatory and immunomodulatory mediators (e.g., chemokines such as CCL1, CCL2, and CCL5) compared with PPP [17].

We hypothesized that the high concentration of CCL1 in L-PRP promotes binding to CCR8 on senescent fibroblasts, triggering activation and structural modulation of PKM2. This signaling axis may facilitate the formation of PKM2 dimers, which readily undergo nuclear translocation due to their structural flexibility. Once in the nucleus, PKM2 dimers activate the JAK/STAT3 pathway, upregulating anti-apoptotic markers (Bcl-2, Bcl-xL) and proliferative factors (Cyclin D1) to rescue fibroblasts from growth arrest. In contrast, the tetrameric form of PKM2 primarily remains in the cytoplasm, where it interacts with SMAD7. This interaction neutralizes SMAD7-mediated inhibition of TGF-β signaling, restoring the collagen synthesis capacity of aged fibroblasts. To validate this dual mechanism, we utilized H_2_O_2_-induced senescent human dermal fibroblasts (HDFs) for in vitro mechanistic studies; we also performed intradermal injections of L-PRP in aged mice to compare their effects on in vivo collagen production.

## 2. Results

### 2.1. L-PRP Increases CCL1-CCR8 Binding, NuclearPKM2 Dimer Signaling, and Proliferation Markers in Senescent Fibroblasts

**Figure 1:**
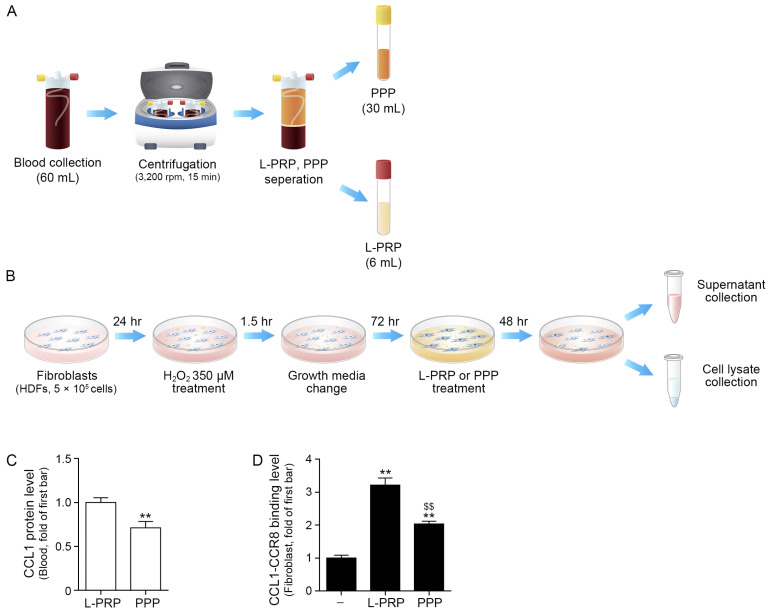
Preparation of L-PRP and PPP and evaluation of L-PRP-mediated CCL1-CCR8 binding in H_2_O_2_-induced senescent HDFs. (**A**) Schematic illustration of L-PRP and PPP preparation from peripheral blood using an automated platelet separation procedure. (**B**) Experimental scheme for the establishment of H_2_O_2_-induced senescent HDFs and subsequent treatment with 5% (*v*/*v*) L-PRP or PPP. (**C**) CCL1 protein levels in 5% (*v*/*v*) L-PRP and PPP preparations, expressed as fold change relative to the first bar. (**D**) CCL1-CCR8 binding levels in H_2_O_2_-induced senescent HDFs treated with 5% (*v*/*v*) L-PRP or PPP, expressed as fold of the first bar. Data are shown as the mean ± standard deviation (*n* = 5). Statistical comparisons were performed using the Kruskal–Wallis test followed by the Mann–Whitney U test; ** *p* < 0.01, vs. first bar; $$ *p* < 0.01, vs. second bar. h, hour; HDFs, human dermal fibroblasts; H_2_O_2_, hydrogen peroxide; L-PRP, leukocyte-rich platelet-rich plasma; PPP, platelet-poor plasma.

L-PRP and PPP were prepared from peripheral blood using an automated platelet separation procedure (Figure 1A). H_2_O_2_-induced senescent HDFs were subsequently treated with L-PRP or PPP after senescence induction (Figure 1B). To determine the optimal concentration of L-PRP for subsequent experiments, we first assessed its cytotoxicity and dose-dependent biological effects in H_2_O_2_-treated HDFs. As shown in Figure S1A, L-PRP did not induce significant cell death at concentrations up to 5%, whereas a slight increase in cytotoxicity was observed at 10%. Evaluation of cell proliferation and receptor-binding efficacy demonstrated that L-PRP significantly increased fibroblast proliferation in a dose-dependent manner up to 5% (Figure S1B). Notably, the proliferation rate and CCL1-CCR8 binding levels both reached a plateau at 5%; no significant differences were observed at higher concentrations (Figure S1C). Accordingly, 5% L-PRP was selected as the optimal concentration for all subsequent in vitro mechanistic studies.

We then compared the efficacy of L-PRP with PPP at the standardized 5% concentration. Enzyme-linked immunosorbent assay (ELISA) results showed that L-PRP contained significantly higher levels of CCL1 relative to PPP (Figure 1C). This was associated with a significantly greater degree of CCL1-CCR8 binding in L-PRP-treated cells than in PPP-treated cells (Figure 1D).

**Figure 2:**
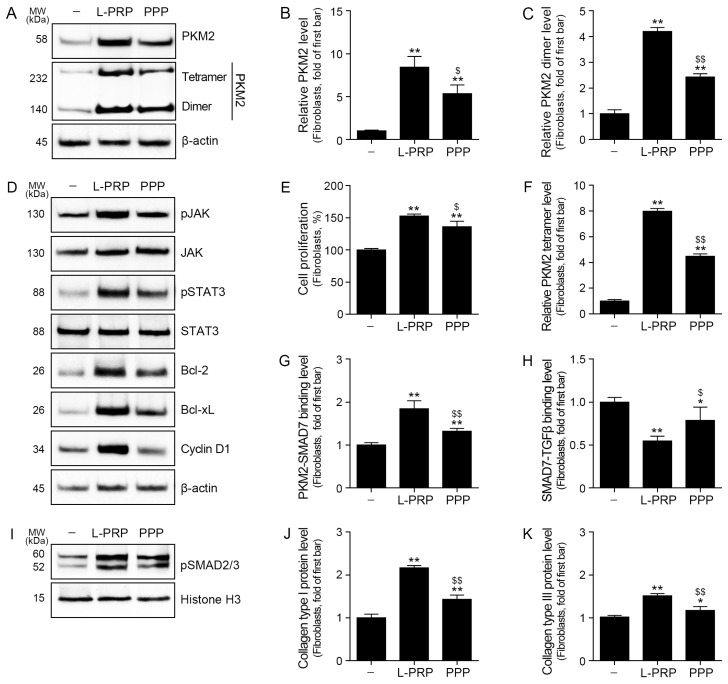
L-PRP promotes PKM2-associated proliferative and collagen synthetic singling in H_2_O_2_-induced senescent HDFs. (**A**) Western blot analysis of total PKM2, PKM2 dimer, and PKM2 tetramer levels in H_2_O_2_-induced senescent HDFs treated with 5% (*v*/*v*) L-PRP and PPP. (**B**,**C**) Quantification of total PKM2 and PKM2 dimer levels normalized to β-actin and expressed as fold change relative to the first bar. (**D**) Western blot analysis of JAK/STAT3 phosphorylation and downstream pro-survival and proliferative proteins, including Bcl-2, Bcl-xL, and Cyclin D1. (**E**) Cell proliferation rate in H_2_O_2_-induced senescent HDFs treated with 5% (*v*/*v*) L-PRP and PPP. (**F**) Quantification of PKM2 tetramer levels from panel A, normalized to β-actin and expressed as fold change relative to the first bar. (**G**,**H**) PKM2-SMAD7 and SMAD7-TGF-β binding levels, expressed as fold change relative to the first bar. (**I**) Western blot analysis of nuclear pSMAD2/3 levels. (**J**,**K**) Collagen type I (**J**) and collagen type III (**K**) protein levels, expressed as fold change relative to the first bar. Data are shown as the mean ± standard deviation (*n* = 5). Statistical comparisons were performed using the Kruskal–Wallis test followed by the Mann–Whitney U test; * *p* < 0.05 and ** *p* < 0.01, vs. first bar; $ *p* < 0.05 and $$ *p* < 0.01, vs. second bar. HDFs, human dermal fibroblasts; H_2_O_2_, hydrogen peroxide; L-PRP, leukocyte-rich platelet-rich plasma; PPP, platelet-poor plasma.

Compared with untreated control and PPP, L-PRP increased total PKM2 expression and promoted PKM2 dimer abundance in H_2_O_2_-induced senescent fibroblasts (Figure 2A–C). L-PRP also increased the phosphorylation ratios of JAK and STAT3 relative to total protein levels (Figure 2D and Figure S2A,B). Consistent with these findings, L-PRP elevated levels of STAT3-associated pro-survival and proliferative proteins, including Bcl-2, Bcl-xL and cyclin D1, compared with PPP (Figure 2D and Figure S2C–E). Furthermore, L-PRP was significantly more effective than PPP in promoting proliferation of H_2_O_2_-induced senescent HDFs (Figure 2E).

### 2.2. L-PRP Increases PKM2 Tetramer Formation and SMAD Signaling Associated with Collagen Synthesis in Senescent Fibroblasts

L-PRP increased PKM2 tetramer levels compared with control and PPP in H_2_O_2_-induced senescent fibroblasts (Figure 2A,F). L-PRP also increased PKM2-SMAD7 binding and decreased SMAD7-TGF-β binding (Figure 2G,H). Consistent with reduced SMAD7-mediated inhibition of TGF-β signaling, nuclear pSMAD2/3 levels increased with L-PRP relative to PPP (Figure 2I and Figure S2F). Protein levels of collagens I and III were elevated with L-PRP relative to PPP in senescent fibroblasts (Figure 2J,K).

### 2.3. L-PRP Dose-Dependently Increases CCL1-CCR8 Binding, NuclearPKM2 Dimer Signaling, and Proliferation Markers in the Aged Skin

**Figure 3:**
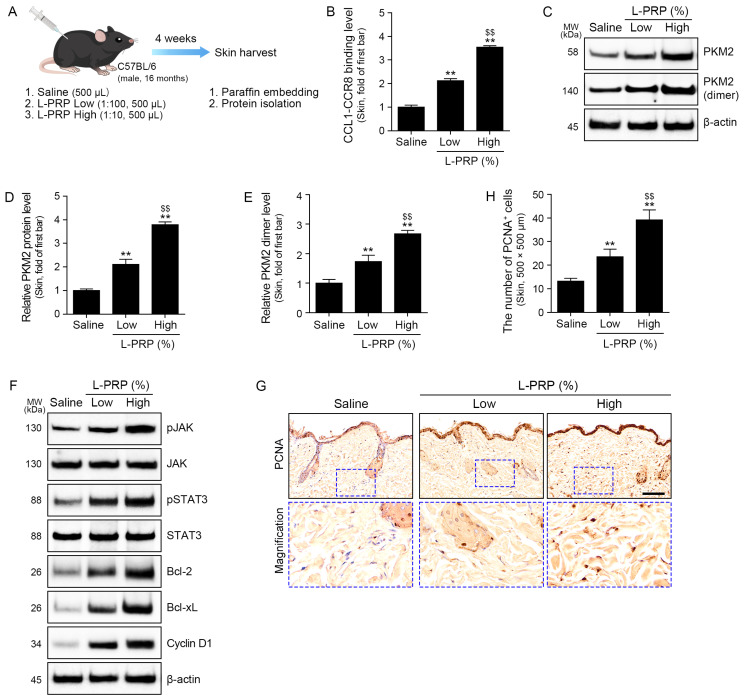
L-PRP dose dependently enhance CCL1-CCR8 binding, and PKM2 dimer-associated proliferative signaling in aged mouse skin. (**A**) Experimental scheme for in vivo L-PRP injection. Sixteen-month-old C57BL/6 male mice were assigned to the Aging/Saline, Aging/L-PRP Low (1:100 dilution), or Aging/L-PRP High (1:10 dilution) group. Saline or diluted L-PRP was administered intradermally into the dorsal skin at a total volume of 500 μL, and dorsal skin samples were collected 4 weeks after injection. (**B**) CCL1-CCR8 binding levels in skin tissues, expressed as fold change relative to the first bar. (**C**) Western blots analysis of total PKM2 and PKM2 dimer levels in skin tissue treated with saline or L-PRP. (**D**,**E**) Quantification of total PKM2 and PKM2 dimer levels normalized to β-actin and expressed as fold change relative to the first bar. (**F**) Western blot analysis of JAK, pJAK, STAT3, pSTAT3, Bcl-2, Bcl-xL, and Cyclin D1 in aged mouse skin treated with saline and L-PRP. (**G**) Representative immunohistochemical staining images of the proliferation marker PCNA in dorsal skin from each group. Brown color indicates PCNA-positive signals and blue color indicates nuclei; scale bar, 100 μm. (**H**) Quantification of PCNA-positive cells number (cells/500 μm × 500 μm). Data are presented as mean ± standard deviation (*n* = 5). Statistical comparisons were performed using the Kruskal–Wallis test followed by the Mann–Whitney U test; ** *p* < 0.01, vs. first bar; $$ *p* < 0.01, vs. second bar. L-PRP, leukocyte-rich platelet-rich plasma; PPP, platelet-poor plasma.

Given that PPP exhibited significantly lower efficacy than L-PRP during the initial in vitro assays, it was excluded from subsequent animal studies. Thus, in vivo experiments focused on evaluating the dose-dependent effects of L-PRP by administering low and high concentrations (Figure 3A). In 16-month-old mouse skin, L-PRP increased CCL1-CCR8 binding in a dose-dependent manner compared with saline-treated skin (Figure 3B). In parallel, L-PRP increased total PKM2 expression and PKM2 dimer levels (Figure 3C–E and Figure S3). L-PRP also enhanced JAK/STAT3 phosphorylation and increased the Bcl-2, Bcl-xL, and cyclin D1 levels in L-PRP-treated skin compared with saline controls (Figure 3F and Figure S4). Proliferation marker staining (proliferating cell nuclear antigen [PCNA]) was increased in L-PRP-treated aged skin, and quantification demonstrated a dose-dependent response (Figure 3G,H).

### 2.4. L-PRP Increases PKM2 Tetramer Formation, SMAD Activation, Collagen Deposition, and Tissue-Level Elasticity in Aged Skin

**Figure 4:**
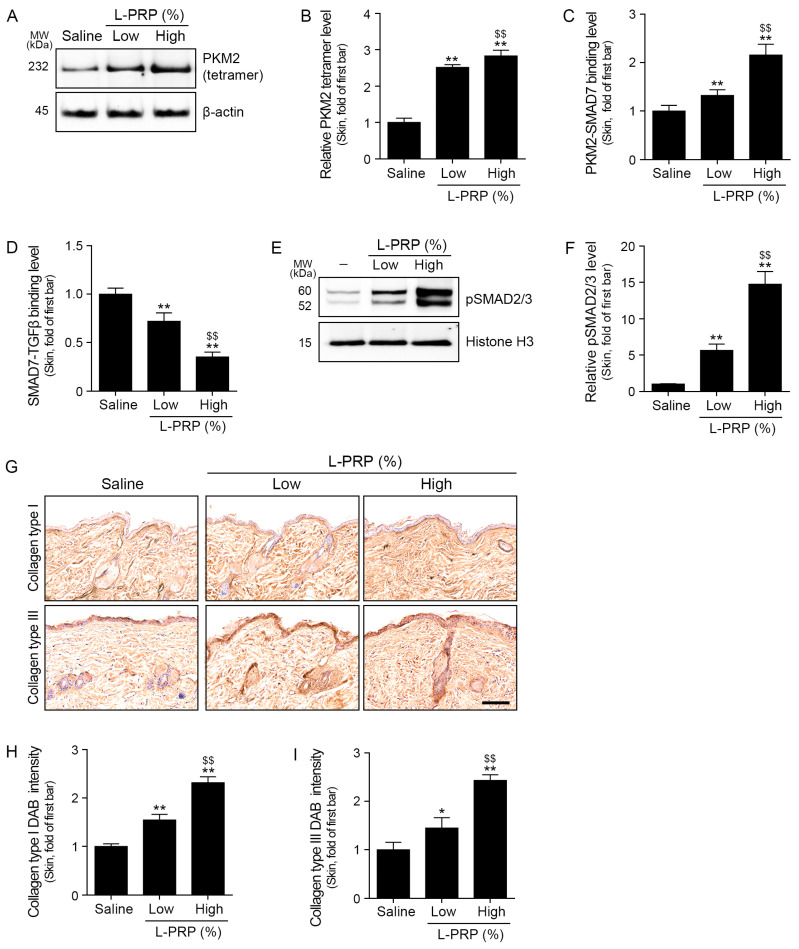
L-PRP enhances PKM2 tetramer formation, SMAD activation, and collagen deposition in aged mouse skin. (**A**) Representative Western blots of PKM2 tetramer and β-actin in dorsal skin from 16-month-old mice treated with low dose L-PRP (L-PRP Low; 1:100 dilution), or high dose L-PRP (L-PRP High; 1:10 dilution). (**B**) Quantification of PKM2 tetramer protein levels, normalized to β-actin and expressed as fold change relative to the first bar. (**C**,**D**) PKM2-SMAD7 and SMAD7-TGF-β binding levels, binding levels expressed as fold change relative to the first bar. (**E**) Representative nuclear Western blots of pSMAD2/3 levels. (**F**) Quantification of nuclear pSMAD2/3 level was normalized to histone H3 and expressed as fold change relative to the first bar. (**G**) Representative immunohistochemical staining for collagens type I and III in dorsal skin from saline and L-PRP-treated aged mice. Brown staining indicates collagen type I- or collagen type III-stained fibers, and blue staining indicates nuclei; scale bar, 100 μm. (**H**,**I**) Quantification of collagen type I (**H**) and collagen type III (**I**) staining intensity and expressed as fold change relative to the first bar. Data are presented as mean ± standard deviation (*n* = 5). Statistical comparisons were performed using the Kruskal–Wallis test followed by the Mann–Whitney U test; * *p* < 0.05 and ** *p* < 0.01, vs. first bar; $$ *p* < 0.01, vs. second bar. L-PRP, leukocyte-rich platelet-rich plasma; PPP, platelet-poor plasma.

**Figure 5:**
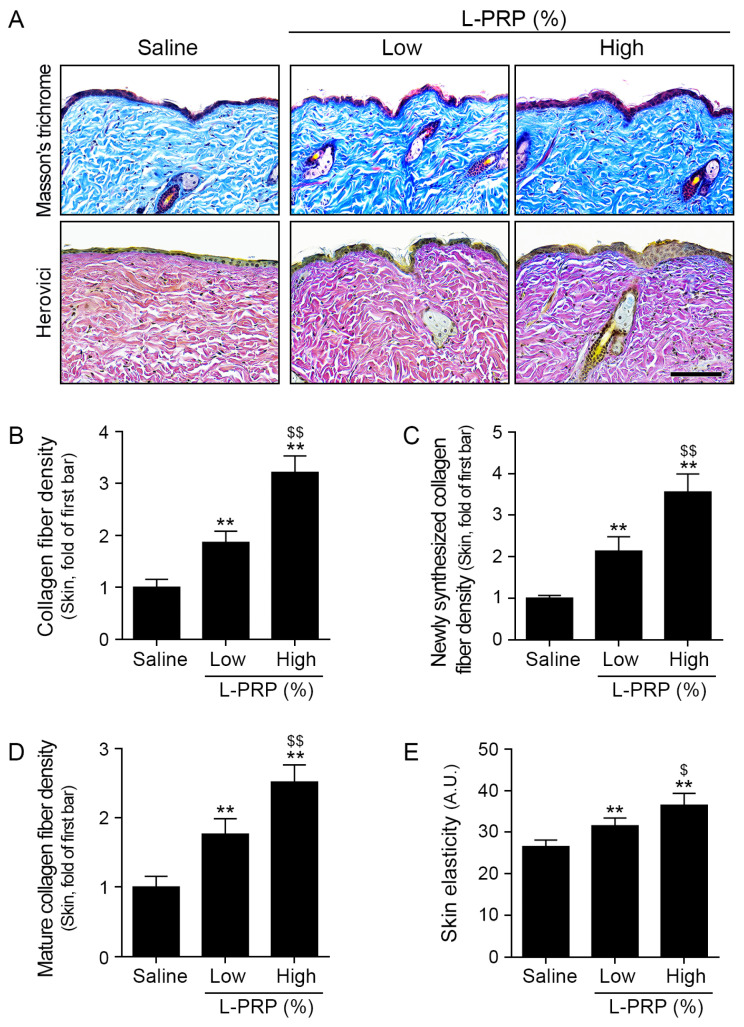
L-PRP increases dermal collagen fiber density and skin elasticity in aged mouse skin. (**A**) Representative Masson’s trichrome (upper panels) and Herovici (lower panels) staining of dorsal skin from 16-month-old mice injected with saline, low dose L-PRP (L-PRP Low; 1:100 dilution), or high dose L-PRP (L-PRP High; 1:10 dilution). In Masson’s trichrome staining, collagen fibers are stained blue. In Herovici staining, newly synthesized collagen fibers are stained blue, whereas mature collagen fibers stained red; scale bar, 100 μm. (**B**) Quantification of total collagen fiber density based on Masson’s trichrome staining. (**C**) Quantification of newly synthesized collagen fiber density from Herovici staining. (**D**) Quantification of mature collagen fiber density (fold of first bar) from Herovici staining. Values are expressed as fold change relative to the first bar. (**E**) Skin elasticity (A.U.) measured using a noninvasive skin analyzer in saline or L-PRP-treated aged mice. Data are presented as mean ± standard deviation (*n* = 5). Statistical comparisons were performed using the Kruskal–Wallis test followed by the Mann–Whitney U test; ** *p* < 0.01, vs. first bar; $ *p* < 0.05 and $$ *p* < 0.01, vs. second bar. A.U. arbitrary units; L-PRP, leukocyte-rich platelet-rich plasma; PPP, platelet-poor plasma.

In aged mouse skin, L-PRP increased PKM2 tetramer levels and enhanced PKM2-SMAD7 binding while decreasing SMAD7-TGF-β binding in a dose-dependent manner (Figure 4A–D and Figure S3). Nuclear pSMAD2/3 levels were increased in L-PRP-treated aged skin relative to saline controls (Figure 4E,F). The staining intensity of collagen type I and III increased upon L-PRP treatment, consistent with enhanced dermal collagen deposition (Figure 4G–I). Masson’s trichrome and Herovici staining demonstrated a significant, dose-dependent increase in collagen fiber density within L-PRP-treated groups relative to saline controls (Figure 5A,B). Specifically, L-PRP treatment promoted accumulation of newly synthesized and mature collagen fibers (Figure 5A,C,D). Skin elasticity, measured using a skin analyzer, was increased in L-PRP-treated aged mice compared with saline controls (Figure 5E).

## 3. Discussion

PRP has been widely used across regenerative indications, including dermatologic applications, based on the prevailing mechanistic paradigm that concentrated platelets release growth factors and cytokines (e.g., platelet-derived growth factor, epidermal growth factor, fibroblast growth factor, insulin-like growth factors, vascular endothelial growth factor, and TGF-β) that promote fibroblast chemotaxis, proliferation, angiogenesis, and ECM remodeling [12]. In esthetic dermatology, clinical and translational studies suggest that intradermal PRP improves the clinical parameters of photoaged skin; PRP is frequently used as an adjunct to procedures such as microneedling for dermal remodeling [13,14]. Nevertheless, PRP outcomes remain heterogeneous, largely because PRP is not a standardized product; preparation methods yield formulations that differ in terms of platelet concentration, leukocyte content, fibrin architecture, and soluble mediator composition [15]. Although PPP is commonly used as a comparator or control, it may retain biologically active plasma proteins and residual platelets. Activated PPP reportedly influences dermal fibroblast behavior and ECM-related outputs in vitro, indicating that it may possess inherent biological activity rather than serving as a strictly inert baseline [17,30].

A major source of PRP variability is leukocyte content. L-PRP incorporates neutrophils, monocytes, and lymphocytes, which can substantially reshape the injected secretome by adding inflammatory and immunomodulatory cytokines and chemokines [15,16]. Comparative studies in noncutaneous tissues indicate that leukocyte enrichment can increase proinflammatory cytokine levels and may enhance or counteract regenerative outcomes, depending on the microenvironment and target cell state [16,18]. Accordingly, although much of the dermatologic PRP literature has emphasized platelet-derived growth factors, L-PRP may exert additional effects through immune–chemokine signaling that directly influences stromal cell fate [16,31]. Although leukocytes are sometimes considered undesirable due to their proinflammatory potential, accumulating evidence suggests that leukocyte-derived mediators can play context-dependent regenerative roles, particularly in tissues where immune–stromal crosstalk is critical for repair. Nevertheless, the molecular mechanisms by which L-PRP exerts regenerative effects in aged skin remain poorly defined.

Most of the literature concerning PRP and PPP has primarily focused on the role of growth factors in skin rejuvenation. The present study explores an alternative perspective by investigating the CCL1-CCR8 chemokine receptor axis and the metabolic enzyme PKM2 as potential mediators of L-PRP activity in aged skin and senescent fibroblasts.

CCR8 is a CC chemokine receptor with CCL1 as a well-established ligand; CCR8 signaling has been linked to immune cell trafficking and inflammatory programs that interact with tissue remodeling [19]. In fibrotic disease models, CCL1-CCR8 signaling has been implicated in macrophage recruitment and M2 polarization. Macrophage-derived CCL1 can act on CCR8 expressed by mesenchymal effector cells (e.g., hepatic stellate cells), promoting fibrogenic activation via pathways that include JAK/STAT signaling [20,26]. More broadly, stromal–immune crosstalk is regarded as a key determinant of fibroblast activation states; immune-derived mediators (e.g., chemokines) regulate fibroblast proliferation, survival, and matrix production [31].

In the present study, L-PRP contained higher concentrations of CCL1 compared with PPP and induced stronger CCL1-CCR8 binding in senescent fibroblasts, accompanied by increased nuclearPKM2 (dimer). Similarly, intradermal administration of low- and high-dose L-PRP led to increased CCL1-CCR8 binding and nuclear PKM2 (dimer) levels in a dose-dependent manner in aged mice.

PKM2 functions in distinct oligomeric states. In its typical role, it exists as a high-activity tetramer that drives glycolytic flux in the cytosol. It can also exist as lower-activity dimers or monomers [4,32,33,34]. These forms facilitate nonmetabolic signaling, including nuclear translocation and transcriptional regulation or protein kinase activity [14,23,24,25]. Because the dimeric state is less efficient with regard to pyruvate processing, it permits diversion of glycolytic intermediates toward biosynthetic pathways that support cell growth and proliferation [4,32,33,34]. In contrast, the tetrameric form is enzymatically more active and less likely to translocate to the nucleus. However, even in the cytosol, tetrameric PKM2 can participate in signaling complexes that influence fibrotic pathways [4,32,33,34].

In our in vitro senescent fibroblast model, L-PRP increased phosphorylation of JAK and STAT3. This pattern is consistent with the concept that receptor-coupled signals can converge on PKM2 and STAT3; nuclear PKM2 reportedly interacts with transcription factors such as STAT3 and supports STAT3-dependent transcriptional programs [35]. STAT3 activation is well known to promote cell survival and proliferation by inducing anti-apoptotic genes (including Bcl-2 and Bcl-XL) and cell cycle regulators such as cyclin D1 [36]. Consistent with these effects, L-PRP increased Bcl-2, Bcl-xL, and cyclin D1 levels and enhanced fibroblast proliferation relative to PPP in our study.

These observations are biologically relevant to dermal aging because aged skin exhibits reduced fibroblast functional capacity and disrupted homeostasis. Senescent fibroblasts display impaired proliferative potential and contribute to ECM loss [4,37]. Chronologically aged human skin reportedly exhibits reduced fibroblast numbers and diminished collagen production capacity, contributing to dermal thinning and wrinkle formation [4,37]. In this context, an intervention that restores survival and proliferative signaling in senescent fibroblasts could expand the pool of matrix-producing cells and support ECM re-accumulation, provided that fibrosis or scarring is not excessively induced [4].

Importantly, the present study demonstrates an association—rather than definitive causality—between CCR8 engagement and PKM2 dimer formation. We did not directly assess whether CCR8 activation is necessary and sufficient for the observed increase in nuclear PKM2 (dimer) under L-PRP conditions. To establish causality, future studies should incorporate functional perturbations such as CCL1 neutralization or depletion in L-PRP, CCR8 blockade or antagonism, or CCR8 knockdown or knockout in fibroblasts, followed by direct assessment of PKM2 oligomeric state and nuclear translocation. These approaches would clarify whether CCL1-CCR8 signaling acts upstream of PKM2 dimerization in this context or whether both are co-regulated by another L-PRP-induced pathway.

A second major finding of our study is that L-PRP increased PKM2 (tetramer) and shifted SMAD7-associated complexes toward enhanced TGF-β signaling in fibroblasts and aged skin. SMAD7 is a canonical inhibitory SMAD that negatively regulates TGF-β receptor signaling; disruption of SMAD7-mediated inhibition can amplify TGF-β/SMAD2/3 activity and fibrotic ECM transcription [4].

Recent mechanistic studies indicate that PKM2 tetramer can directly interact with SMAD7 and interfere with SMAD7-mediated negative regulation of TGF-β signaling, thus enhancing downstream signaling and fibrosis [4]. Consistent with this model, L-PRP increased PKM2 tetramer levels, enhanced PKM2-SMAD7 binding, reduced SMAD7-TGF-β binding, and increased pSMAD2/3 levels. These changes coincided with increased expression of collagens I and III in vitro and enhanced dermal collagen deposition in vivo.

A key mechanistic gap revealed by these data concerns how L-PRP increases PKM2 tetramer levels. Although emerging evidence suggests that CCL1-CCR8 signaling can promote PKM2 induction, there is limited direct evidence that this pathway specifically drives PKM2 tetramerization, rather than PKM2 expression or dimer-associated nuclear functions. Furthermore, our data do not clarify whether CCL1-CCR8 signaling directly promotes PKM2 tetramerization. Accordingly, the tetramer arm of this model should be considered an empirically supported downstream effect of L-PRP treatment with an unresolved proximal mechanism.

Mechanistic analyses indicate that PKM2 oligomerization is regulated by multiple inputs, including allosteric metabolites (e.g., fructose-1,6-bisphosphate), post-translational modifications (phosphorylation, acetylation, and oxidation), and protein–protein interactions. These factors suggest several plausible mechanisms by which L-PRP may promote PKM2 tetramer formation [38].

Our animal data demonstrate a dose-dependent pattern, in which high-dose L-PRP more strongly increased proliferative signaling (PCNA), ECM signaling (pSMAD2/3), and structural outcomes (collagen fiber and elasticity) compared with low-dose L-PRP. This dose–response relationship supports biological plausibility and conflicts with a purely nonspecific injection effect, although it leaves unresolved the components of L-PRP (platelets, leukocytes, or plasma factors) that predominantly drive each signaling pathway.

Previous studies have shown that leukocyte content can influence whether PRP promotes inflammation or tissue repair, depending on the microenvironment [16]. Our findings suggest that CCL1, a chemokine associated with leukocytes, can serve as a key parameter for optimizing PRP formulations intended to restore the dermal matrix in older patients.

This study has several limitations. First, a direct cause–effect relationship between CCL1-CCR8 engagement and PKM2 dimer formation or STAT3 activation was not established; future loss-of-function studies (e.g., CCL1 neutralization or silencing, CCR8 blockade) are required. Second, the upstream mechanism underlying PKM2 tetramer accumulation after L-PRP treatment remains undefined, and further investigation of PKM2 oligomer regulation in senescent fibroblasts is needed. Third, human L-PRP was administered to mice. Although the consistent dose-dependent effects suggest that xenogeneic inflammation did not substantially confound the findings, species-specific immune responses cannot be excluded. Future studies should incorporate matched mouse-derived PRP controls. Finally, this work should be regarded as a mechanistic proof of concept demonstrating that L-PRP can engage a PKM2-linked pathway to promote ECM restoration in an aging context, rather than definitive evidence of clinical anti-aging efficacy, which will require controlled human studies with standardized PRP characterization. Finally, in this study, leukocyte-high and leukocyte-low PRP preparations were not directly compared. In the present in vivo experiments, the low- and high-dose groups represented different dilutions of the same L-PRP preparation rather than PRP formulations with different leukocyte contents. Therefore, although our data support the biological activity of L-PRP, they do not determine the specific contribution of leukocyte abundance to the observed effects. Future studies should compare leukocyte-rich and leukocyte-poor PRP preparations with matched platelet concentrations and standardized PRP characterization.

Despite these limitations, the findings expand the mechanistic framework through which L-PRP may promote dermal ECM regeneration. Beyond the conventional growth factor-centric paradigm, our data supports a model in which L-PRP enhances ECM production by (i) promoting fibroblast survival and proliferation through a CCL1-CCR8-associated PKM2 (dimer) JAK/STAT3 pathway and (ii) amplifying TGF-β/SMAD2/3 signaling via PKM2 (tetramer) interactions with SMAD7.

Conceptually, these results position L-PRP as an immuno-regenerative intervention that can modulate senescent fibroblast fate via chemokine–metabolic coupling. This framework may help explain why leukocyte content substantially influences PRP responses; it suggests that mediator-guided PRP formulation, or combination strategies targeting CCR8 or PKM2, could represent future approaches for skin rejuvenation in aging.

## 4. Materials and Methods

### 4.1. Study Participants and Ethical Approval

This study was reviewed and approved by the Public Institutional Review Board designated by the Ministry of Health and Welfare of Korea (approval no. P01-202507-02-011). The study was conducted in accordance with the Declaration of Helsinki and relevant Korean bioethics regulations for human-derived materials. Written informed consent was obtained from all participants prior to blood collection. A total of five eligible participants were enrolled in this study. Eligible participants were adults aged 20–65 years who voluntarily agreed to donate peripheral venous blood for this study. Individuals were excluded if they had a history of anemia, were currently taking antiplatelet or anticoagulant medications, had a previous history of syncope after blood collection, had skin disease at peripheral blood collection sites such as the arms or legs, or were pregnant. Participant confidentiality was maintained throughout the study, and no personally identifiable information was used in subsequent analyses.

### 4.2. Blood Collection

Peripheral venous blood was collected under sterile conditions using a standard venipuncture procedure. For each participant, 52 mL of whole blood was collected and mixed with 8 mL of acid–citrate–dextrose solution A (ACD-A) as an anticoagulant. The anticoagulated blood mixture was immediately processed for PRP preparation using the GPS^®^ III Platelet Concentration System. To minimize risks associated with blood collection, participant identity and general condition were checked before venipuncture, an appropriate peripheral vein was selected, and standard aseptic procedures were followed. After blood collection, adequate compression was applied to the puncture site, and participants were monitored for possible adverse events such as hematoma, bleeding, dizziness, vasovagal reaction, or other rare complications.

### 4.3. Preparation of L-PRP and PPP Using an Automated Platelet Concentration System

L-PRP and PPP were prepared using a commercially available automated platelet concentration system (GPS^®^ III Platelet Concentration System; Zimmer Biomet, Warsaw, IN, USA) according to the manufacturer’s instructions. Briefly, 52 mL of whole blood was collected and mixed with 8 mL ACD-A. The anticoagulated whole blood was then transferred into the GPS^®^ III separation tube and centrifuged at 3200 rpm for 15 min. After centrifugation, the blood components were separated into distinct layers, including a lower red blood cell layer, an intermediate buffy coat layer corresponding to L-PRP, and an upper plasma layer corresponding to PPP. The PPP fraction was first aspirated from the upper plasma layer, and the L-PRP fraction was subsequently obtained from the buffy coat-containing layer. The obtained L-PRP and PPP were transferred into sterile tubes containing 1% bovine serum albumin in phosphate-buffered saline (PBS), stored at 4 °C, and used for subsequent analyses and in vivo experiments within 3 h of preparation.

### 4.4. In Vivo Study

#### 4.4.1. Animal Housing and Ethics Statement

Male C57BL/6 mice obtained from Orient Bio (Seongnam, Republic of Korea) were housed under standard laboratory conditions at 20–24 °C and 45–55% humidity, with a 12 h light/dark cycle and free access to food and water. After a 1-week acclimation period, mice were used at 16 months of age as an aging model. All animal procedures were conducted in accordance with the guidelines of the Institutional Animal Care and Use Committee of Gachon University (IACUC no. LCDI-2025-0073) and complied with the ethical standards of AAALAC International (Frederick, MD, USA) and the ARRIVE guidelines.

#### 4.4.2. Experimental Design and L-PRP Administration

Aged mice (16 months old) were randomly allocated into three groups: Aging/Saline, Aging/L-PRP (Low; 1:100 dilution), and Aging/L-PRP (High; 1:10 dilution) (*n* = 5 per group). Each mouse served as an experimental unit. All injections and evaluations were performed by investigators who had been blinded to group allocation to minimize bias. No animals met the predefined exclusion criteria (e.g., severe intercurrent illness, substantial weight loss, or unexpected adverse events), and all animals completed the study.

For all procedures, mice were anesthetized with inhalational isoflurane. The dorsal skin was shaved and disinfected with 70% ethanol prior to injection. In the Aging/Saline group, 500 µL of sterile saline was intradermally injected into the dorsal dermis. In the Aging/L-PRP (Low) group, L-PRP was diluted 1:100 in saline, and 500 µL of diluted L-PRP were intradermally injected into the dorsal dermis. In the Aging/L-PRP (High) group, L-PRP was diluted 1:10 in saline, and 500 µL of the diluted L-PRP was intradermally injected into the same region. In all groups, the total volume was delivered as multiple small intradermal blebs evenly distributed across the dorsal area.

Animals were evaluated 4 weeks after injection. At the endpoint, full thickness dorsal skin samples encompassing the injection sites were harvested for molecular and histological analyses. Histological evaluation and quantitative image analysis were performed by investigators who had been blinded to treatment group to minimize observer bias.

#### 4.4.3. Skin Elasticity Measurement

Skin elasticity was assessed using an API 100 Skin Analyzer (Aram Huvis, Seongnam, Republic of Korea). High-resolution images of the skin surface were captured using a noncontact optical method, and elasticity was quantified using the accompanying software (Solutionist v1.7.10). Each mouse was measured five times immediately prior to sampling, and the average value was calculated.

### 4.5. In Vitro Experiments

#### 4.5.1. Cell Culture

HDFs were purchased from CEFO Bio (Seoul, Republic of Korea) and cultured using the CEFO™ Human Dermal Fibroblast Cell Kit, in accordance with the manufacturer’s instructions. The culture medium was replaced every 2–3 days. Cells were maintained at 37 °C in a humidified atmosphere containing 5% CO_2_. Cells between passages 4 and 7 were used for all experiments; all experiments were performed in triplicate.

#### 4.5.2. Induction of Cellular Senescence and L-PRP/PPP Treatment in HDFs

Cellular senescence in HDFs was induced by treatment with 350 μM H_2_O_2_ for 1.5 h, followed by medium replacement and 72 h of culture [39]. Senescent HDFs were used for all subsequent experiments. L-PRP and PPP were prepared from human peripheral blood as described above and diluted in culture medium immediately before use.

For cytotoxicity assessment, senescent HDFs were seeded in 96-well plates at a density of 5 × 10^4^ cells/well and treated with L-PRP at final concentrations of 0%, 1%, 2%, 5%, or 10% (*v*/*v*) for 48 h. Cell viability was evaluated using cell counting kit (CCK)-8, in accordance with the manufacturer’s instructions. To determine the optimal L-PRP concentration, senescent HDFs were treated with 0%, 2.5%, 5%, or 7.5% (*v*/*v*) L-PRP for 48 h under the same conditions; 5% (*v*/*v*) L-PRP was identified as the optimal concentration, providing maximal efficacy without detectable cytotoxicity.

Based on these results, all subsequent experiments were performed using 5% (*v*/*v*) L-PRP. For comparison at an equivalent volume fraction, PPP was also diluted to a final concentration of 5% (*v*/*v*) in culture medium. In all experiments, L-PRP or PPP was added 72 h after H_2_O_2_ treatment. Senescent HDFs were incubated with 5% (*v*/*v*) L-PRP or PPP for 48 h prior to sample collection for molecular and functional analyses.

### 4.6. Cell Death and Proliferation Assays

Cell proliferation and viability were assessed via CCK-8 (TransGen Biotech, Beijing, China). For cytotoxicity assessment, senescent HDFs were seeded at a density of 5 × 10^4^ cells/well and treated with L-PRP at concentrations of 0%, 1%, 2%, 5%, or 10% (*v*/*v*) for 48 h. For proliferation assays, senescent HDFs were seeded in 96-well plates at a density of 1 × 10^4^ cells/well and allowed to attach overnight, then treated with L-PRP at final concentrations of 0%, 2.5%, 5%, or 7.5% (*v*/*v*) for 48 h to determine the optimal concentration, as described above. In all experiments, CCK 8 solution was added to each well for the final 2 h of incubation, and absorbance at 450 nm was measured using a microplate reader. Cell proliferation or viability was expressed as a percentage of the untreated control.

### 4.7. Protein Isolation and Quantification

Tissue and cell samples were lysed using the EzRIPA Lysis Kit (ATTO Corporation, Tokyo, Japan), which contains radioimmunoprecipitation assay buffer supplemented with protease and phosphatase inhibitors (each at 100× dilution). For tissue samples, excised skin tissues were washed once with PBS, and lysis buffer was added at a ratio of 1 mL per 40 mg of tissue. Samples were incubated on ice at 4 °C for 10 min, then sonicated for 15 min (10 s on, 1 min off cycles). For cell samples, the culture medium was removed, and cells were washed three times with PBS. Lysis buffer (500 μL per 10 cm dish) was added, and cells were incubated on ice at 4 °C for 5 min. Cells were then scraped and disrupted by sonication for 10 min (10 s on, 1 min off cycles). Tissue and cell lysates were centrifuged at 14,000× *g* for 15 min at 4 °C, and supernatants were collected. For nuclear protein extraction, cell lysates were processed via NE-PER™ Nuclear and Cytoplasmic Extraction Reagents (Invitrogen/Thermo Fisher Scientific, Waltham, MA, USA), using the manufacturer’s protocol to separate cytoplasmic and nuclear fractions. Protein concentrations were determined using the BCA Protein Assay Kit (Thermo Fisher Scientific) with bovine serum albumin as the standard. Absorbance was measured at 562 nm using a Multiskan SkyHigh microplate spectrophotometer (Thermo Fisher Scientific). Protein samples were stored at −80 °C until use.

### 4.8. Indirect ELISA

For CCL1 measurement, L-PRP and PPP samples were diluted to a final concentration of 5% (*v*/*v*) in carbonate–bicarbonate coating buffer (pH 9.6), and 96 well plates (SPL Life Sciences, Pocheon, Republic of Korea) were coated with the diluted samples by overnight incubation at 4 °C. After plates had been coated, they were washed three times with PBS containing 0.1% Tween 20, then blocked with 5% skim milk (LPS Solution, Daejeon, Republic of Korea) in PBS for 1 h at room temperature. A primary antibody against CCL1 (details listed in Table S1) was added to each well and incubated overnight at 4 °C; this was followed by three washes with PBS containing 0.1% Tween 20. Horseradish peroxidase-conjugated secondary antibodies (1:1000; Vector Laboratories, Burlingame, CA, USA) were then applied for 1 h at room temperature. Color development was achieved using 3,3′,5,5′ tetramethylbenzidine (TMB) substrate solution (Sigma Aldrich, St. Louis, MO, USA), and the reaction was terminated with 1 M sulfuric acid. Absorbance was measured at 450 nm using a Multiskan SkyHigh microplate spectrophotometer (Thermo Fisher Scientific). CCL1 levels were expressed as absorbance values relative to the control group.

For collagens I and III, indirect ELISA was performed using cell lysates. Protein samples extracted from HDFs were diluted in carbonate–bicarbonate coating buffer (pH 9.6), adhered to 96-well plates by overnight incubation at 4 °C, and processed as described above using primary antibodies against collagens I and III (Table S1), followed by horseradish peroxidase-conjugated secondary antibodies and TMB substrate. Absorbance at 450 nm was recorded. Collagen levels were normalized to the control group or to total protein content, as indicated in the figure legends.

### 4.9. Sandwich ELISA

Sandwich ELISA was performed to comparatively evaluate target protein levels in cell culture supernatants and skin tissue extracts. Ninety six-well plates were coated with capture antibodies specific for the target proteins diluted in carbonate–bicarbonate coating buffer (pH 9.6), then incubated overnight at 4 °C. After three washes with PBS containing 0.1% Tween 20, wells were blocked with 5% skim milk in PBS for 1 h at room temperature.

Cell culture supernatants from HDFs or homogenized skin tissue lysates were added to the wells and incubated for 24 h at room temperature. This incubation step was followed by washing and further incubation with detection antibodies against the corresponding targets for 24 h. Horseradish peroxidase-conjugated secondary antibodies were then applied for 2 h at room temperature. Color development was achieved using TMB substrate, and the reaction was terminated with 1 M sulfuric acid. Absorbance was measured at 450 nm using a microplate spectrophotometer. Relative protein levels were expressed as absorbance values normalized to the control group.

### 4.10. Western Blot Analysis

Protein samples were denatured with 4× lithium dodecyl sulfate sample buffer and 10× reducing reagent (Thermo Fisher Scientific) at 70 °C for 10 min. Thirty micrograms of total protein per lane were resolved on 10% sodium dodecyl sulfate–polyacrylamide gels using MOPS buffer (Thermo Fisher Scientific) at 200 V for 25 min, in conjunction with appropriate molecular weight markers (EasySee Western Marker and EasySee II Western Marker; TransGen Biotech). Proteins were transferred to polyvinylidene fluoride membranes (Millipore, Burlington, MA, USA) using a semi-dry transfer system (ATTO Corporation) at 1 A for 10 min. Membranes were blocked with 5% skim milk (LPS Solution) in Tris-buffered saline plus Tween (TBST) for 1 h at room temperature, then probed with primary antibodies (Table S1) overnight at 4 °C. After three 10 min washes with TBST, membranes were incubated with horseradish peroxidase-conjugated secondary antibodies (1:2000–1:10,000; Vector Laboratories) for 1 h at room temperature. Following three additional 10 min washes with TBST, immunoreactive bands were visualized using an enhanced chemiluminescence reagent (Cytiva, Marlborough, MA, USA) on a ChemiDoc system (Bio-Rad Laboratories, Hercules, CA, USA). Band intensities were quantified using ImageJ (v1.53s; National Institutes of Health, Bethesda, MD, USA), normalized to histone H3 or β-actin, and expressed as fold change relative to the control group.

### 4.11. Blue Native PAGE (BN-PAGE) Analysis of PKM2 Oligomerization

BN-PAGE was performed to analyze PKM2 oligomerization in L-PRP-treated cells and skin tissues. After 48 h of treatment with 5% (*v*/*v*) L-PRP or PPP, cells were washed twice with cold PBS. Skin tissue from the corresponding treatment groups were collected and homogenized. Cells and tissue homogenates were lysed on ice for 30 min in BN-PAGE lysis buffer (50 mM Bis Tris HCl, 0.5 M 6-aminohexanoic acid, 10% glycerol, and 1% digitonin, pH 7.0) supplemented with protease and phosphatase inhibitors (ATTO Corporation). Lysates were centrifuged at 16,000× *g* for 15 min at 4 °C, and protein concentrations in the supernatants were determined using a BCA Protein Assay Kit (Thermo Fisher Scientific). BN-PAGE loading buffer containing Coomassie brilliant blue G-250 (Sigma-Aldrich) was then added to prepare samples for electrophoresis.

Equal amounts of protein were loaded onto native polyacrylamide gels; electrophoresis was carried out at a constant voltage of 100 V using an inner buffer (0.05 M Tricine, 15 mM Bis Tris, pH 7.0) and an outer buffer (0.05 M Bis Tris HCl, pH 7.0). Proteins were then transferred onto polyvinylidene fluoride membranes at a constant current of 300 mA [40]. After transfer, membranes were briefly destained with methanol, washed twice with TBST, and blocked with 10% skim milk in TBST for 1 h at room temperature. Membranes were incubated overnight at 4 °C with primary antibodies against PKM2, then incubated with horseradish peroxidase-conjugated secondary antibodies and subjected to chemiluminescent detection as described for conventional Western blotting (antibody details are listed in Table S1). Band intensities corresponding to PKM2 tetramers and dimers were quantified using ImageJ software (v1.53s) and expressed relative to the control group.

### 4.12. Histological and Immunohistochemistry Analysis

#### 4.12.1. Tissue Fixation, Processing, and Paraffin Embedding

Skin tissues were fixed in 4% paraformaldehyde (Sigma-Aldrich) at 4 °C for 72 h using a fixative-to-tissue volume ratio of 10:1. Fixed tissues were processed using an automated tissue processor (Tissue-Tek VIP 5 Jr., SAKURA, Tokyo, Japan), in accordance with the manufacturer’s protocol for dehydration, clearing, and paraffin infiltration. Tissues were then embedded in paraffin (Leica, Wetzlar, Germany) using an embedding center (Tissue-Tek TEC, SAKURA). Serial sections (7 μm thick) were cut from paraffin blocks using a microtome (Thermo Fisher Scientific), mounted on coated slides (Muto Pure Chemicals Co., Ltd., Tokyo, Japan), and incubated overnight at 60 °C to ensure adhesion.

#### 4.12.2. Masson’s Trichrome Staining

Paraffin-embedded tissue sections (7 μm) were deparaffinized in xylene and rehydrated through a graded ethanol series (100%, 95%, 90%, 80%, and 70%) to distilled water. Sections were incubated in Bouin’s fluid (ScyTek Laboratories Inc., Logan, UT, USA) at 56–64 °C for 60 min, then incubated at room temperature for 10 min. Sections were rinsed in running tap water for 20 min, then stained with Weigert’s iron hematoxylin (ScyTek Laboratories) for 5 min and rinsed in running tap water for 10 min. Sections were subsequently incubated in Biebrich scarlet/acid fuchsin solution (ScyTek Laboratories) for 2 min, rinsed in running tap water for 10 min, treated with phosphomolybdic/phosphotungstic acid solution (ScyTek Laboratories) for 10 min, and stained with aniline blue solution (ScyTek Laboratories) for 5 min. After they had been rinsed in running tap water for 10 min, sections were differentiated in 1% acetic acid solution for 3 min. Stained sections were dehydrated through a graded ethanol series (95% for 1 min twice, 100% for 1 min twice), cleared in xylene, and mounted.

Images were captured using a slide scanner (Motic Scan Infinity 100; Motic, Hong Kong, China). Collagen content in the dermal area was quantified as the relative intensity of blue-stained regions using ImageJ software (v1.53s). Multiple fields per sample were analyzed.

#### 4.12.3. Herovici Staining

Herovici staining was performed using a commercial Herovici Stain Kit (ScyTek Laboratories), in accordance with the manufacturer’s instructions. Sections were stained with Weigert’s iron hematoxylin followed by Herovici solution, then dehydrated and mounted. Stained slides were scanned using a slide scanner (Motic Scan Infinity 100; Motic), and representative images were captured. The densities of newly synthesized collagen fibers (blue) and mature collagen fibers (red) were separately quantified using ImageJ software (v1.53s). Specifically, young (blue) and mature (red) collagen were separated by color deconvolution [41,42]; the density of each collagen subtype was measured and expressed as fold change relative to the Saline group.

#### 4.12.4. Immunohistochemical Staining

For immunohistochemical staining, paraffin-embedded sections were deparaffinized in xylene and rehydrated through a graded ethanol series. Permeabilization was performed using 0.5% Triton X-100 for 5 min, followed by PBS washes. To block nonspecific binding, sections were incubated with normal serum blocking solution for 1 h at room temperature. Slides were then incubated overnight at 4 °C with primary antibodies (Table S1) diluted in blocking solution. After sections had been washed, they were incubated with biotin-conjugated secondary antibodies (Vector Laboratories) for 1 h at room temperature. Signal amplification was achieved using an avidin–biotin complex reagent (Vector Laboratories), and immunoreactivity was visualized with 3,3′-diaminobenzidine solution (DAB; Sigma-Aldrich) for 5 min to develop a brown reaction product. Nuclei were counterstained with hematoxylin (KPNT, Cheongju, Republic of Korea). Sections were then dehydrated, cleared in xylene, and mounted with DPX mounting medium. Stained tissues were scanned using a Motic Scan Infinity 100 slide scanner (Motic), and representative images were captured. Quantification of DAB-positive staining was performed using ImageJ software (v1.53s) by measuring the number of brown-stained nuclei or density of brown-stained fibers within the dermis. The resulting values were expressed as fold change relative to the control group.

### 4.13. Statistical Analysis

Statistical analyses were performed using SPSS version 26 (IBM Corp., Armonk, NY, USA). All analyses were conducted using nonparametric methods. The Kruskal–Wallis test was used for comparisons among multiple independent groups, followed by the Mann–Whitney U test for pairwise comparisons. Data are presented as mean ± standard deviation. A *p*-value < 0.05 was considered statistically significant. The definitions of statistical symbols used in the graphs are provided in the corresponding figure legends. All experiments were performed in triplicate (*n* = 3 biological replicates), and animal experiments included five animals per group (*n* = 5).

## 5. Conclusions

L-PRP enhances ECM regeneration in aged skin by linking chemokine signaling (CCL1-CCR8) with PKM2 conformational states, thus promoting fibroblast survival and proliferation via JAK/STAT3 while simultaneously amplifying pro-ECM TGF-β/SMAD signaling. These findings provide a mechanistic framework for optimizing PRP formulations beyond platelet-derived growth factor-centered paradigms.

## Data Availability

All data are contained within this article.

## Supplementary Materials

The following supporting information can be downloaded at: https://www.mdpi.com/article/10.3390/ijms27146281/s1.

## References

[1] Farage M.A., Miller K.W., Elsner P., Maibach H.I. (2008). Intrinsic and extrinsic factors in skin ageing: A review. Int. J. Cosmet. Sci..

[2] Lapière C.M. (1990). The ageing dermis: The main cause for the appearance of ‘old’ skin. Br. J. Dermatol..

[3] Kohl E., Steinbauer J., Landthaler M., Szeimies R.M. (2011). Skin ageing. J. Eur. Acad. Dermatol. Venereol..

[4] Varani J., Dame M.K., Rittie L., Fligiel S.E., Kang S., Fisher G.J., Voorhees J.J. (2006). Decreased collagen production in chronologically aged skin: Roles of age-dependent alteration in fibroblast function and defective mechanical stimulation. Am. J. Pathol..

[5] Quan T., Qin Z., Xia W., Shao Y., Voorhees J.J., Fisher G.J. (2009). Matrix-degrading metalloproteinases in photoaging. J. Investig. Dermatol. Symp. Proc..

[6] Talwar H.S., Griffiths C.E., Fisher G.J., Hamilton T.A., Voorhees J.J. (1995). Reduced type I and type III procollagens in photodamaged adult human skin. J. Investig. Dermatol..

[7] Quan T., Little E., Quan H., Qin Z., Voorhees J.J., Fisher G.J. (2013). Elevated matrix metalloproteinases and collagen fragmentation in photodamaged human skin: Impact of altered extracellular matrix microenvironment on dermal fibroblast function. J. Investig. Dermatol..

[8] Quan C., Cho M.K., Perry D., Quan T. (2015). Age-associated reduction of cell spreading induces mitochondrial DNA common deletion by oxidative stress in human skin dermal fibroblasts: Implication for human skin connective tissue aging. J. Biomed. Sci..

[9] Fisher G.J., Shao Y., He T., Qin Z., Perry D., Voorhees J.J., Quan T. (2016). Reduction of fibroblast size/mechanical force down-regulates TGF-β type II receptor: Implications for human skin aging. Aging Cell.

[10] Shin J.W., Kwon S.H., Choi J.Y., Na J.I., Huh C.H., Choi H.R., Park K.C. (2019). Molecular Mechanisms of Dermal Aging and Antiaging Approaches. Int. J. Mol. Sci..

[11] Chicharro-Alcántara D., Rubio-Zaragoza M., Damiá-Giménez E., Carrillo-Poveda J.M., Cuervo-Serrato B., Peláez-Gorrea P., Sopena-Juncosa J.J. (2018). Platelet Rich Plasma: New Insights for Cutaneous Wound Healing Management. J. Funct. Biomater..

[12] Alam M., Hughart R., Champlain A., Geisler A., Paghdal K., Whiting D., Hammel J.A., Maisel A., Rapcan M.J., West D.P. (2018). Effect of Platelet-Rich Plasma Injection for Rejuvenation of Photoaged Facial Skin: A Randomized Clinical Trial. JAMA Dermatol..

[13] Kang C., Lu D. (2022). Combined Effect of Microneedling and Platelet-Rich Plasma for the Treatment of Acne Scars: A Meta-Analysis. Front. Med..

[14] Dohan Ehrenfest D.M., Rasmusson L., Albrektsson T. (2009). Classification of platelet concentrates: From pure platelet-rich plasma (P-PRP) to leucocyte- and platelet-rich fibrin (L-PRF). Trends Biotechnol..

[15] Xu Z., Yin W., Zhang Y., Qi X., Chen Y., Xie X., Zhang C. (2017). Comparative evaluation of leukocyte- and platelet-rich plasma and pure platelet-rich plasma for cartilage regeneration. Sci. Rep..

[16] Devereaux J., Nurgali K., Kiatos D., Sakkal S., Apostolopoulos V. (2018). Effects of platelet-rich plasma and platelet-poor plasma on human dermal fibroblasts. Maturitas.

[17] Korbecki J., Grochans S., Gutowska I., Barczak K., Baranowska-Bosiacka I. (2020). CC Chemokines in a Tumor: A Review of Pro-Cancer and Anti-Cancer Properties of Receptors CCR5, CCR6, CCR7, CCR8, CCR9, and CCR10 Ligands. Int. J. Mol. Sci..

[18] Liu S., Zhang Z., Wang Y., Zhang Y., Min J., Li X., Liu S. (2023). The chemokine CCL1 facilitates pulmonary fibrosis by promoting macrophage migration and M2 polarization. Int. Immunopharmacol..

[19] Diao S., Li L., Zhang J., Ji M., Sun L., Shen W., Wu S., Chen Z., Huang C., Li J. (2025). Macrophage-derived CCL1 targets CCR8 receptor in hepatic stellate cells to promote liver fibrosis through JAk/STAT pathway. Biochem. Pharmacol..

[20] Liu Y., Zhang X., Wang J., Yang F., Luo W., Huang J., Chen M., Wang S., Li C., Zhang W. (2022). ZC3H4 regulates infiltrating monocytes, attenuating pulmonary fibrosis through IL-10. Respir. Res..

[21] Wang Y., Zhang L., Wu G.R., Zhou Q., Yue H., Rao L.Z., Yuan T., Mo B., Wang F.X., Chen L.M. (2021). MBD2 serves as a viable target against pulmonary fibrosis by inhibiting macrophage M2 program. Sci. Adv..

[22] Liu S.S., Liu C., Lv X.X., Cui B., Yan J., Li Y.X., Li K., Hua F., Zhang X.W., Yu J.J. (2021). The chemokine CCL1 triggers an AMFR-SPRY1 pathway that promotes differentiation of lung fibroblasts into myofibroblasts and drives pulmonary fibrosis. Immunity.

[23] Gao W.J., Liu J.X., Liu M.N., Yao Y.D., Liu Z.Q., Liu L., He H.H., Zhou H. (2021). Macrophage 3D migration: A potential therapeutic target for inflammation and deleterious progression in diseases. Pharmacol. Res..

[24] Prasse A., Pechkovsky D.V., Toews G.B., Jungraithmayr W., Kollert F., Goldmann T., Vollmer E., Müller-Quernheim J., Zissel G. (2006). A vicious circle of alveolar macrophages and fibroblasts perpetuates pulmonary fibrosis via CCL18. Am. J. Respir. Crit. Care Med..

[25] Jia S., Shi N., Lu M., Wang X., Qi Y., Wang X., Zhao J., Jiang D. (2025). M2 macrophages promote PKM2 production in fibroblasts to alleviate UVB-induced photoaging. Cell Cycle.

[26] Gao X., Wang H., Yang J.J., Liu X., Liu Z.R. (2012). Pyruvate kinase M2 regulates gene transcription by acting as a protein kinase. Mol. Cell.

[27] Yang W., Xia Y., Ji H., Zheng Y., Liang J., Huang W., Gao X., Aldape K., Lu Z. (2011). Nuclear PKM2 regulates β-catenin transactivation upon EGFR activation. Nature.

[28] Jemal M., Getinet M., Amare G.A., Tegegne B.A., Baylie T., Mengistu E.F., Osman E.E., Chura Waritu N., Adugna A. (2024). Non-metabolic enzyme function of pyruvate kinase M2 in breast cancer. Front. Oncol..

[29] Gao S., Li X., Jiang Q., Liang Q., Zhang F., Li S., Zhang R., Luan J., Zhu J., Gu X. (2022). PKM2 promotes pulmonary fibrosis by stabilizing TGF-β1 receptor I and enhancing TGF-β1 signaling. Sci. Adv..

[30] Kim D.H., Je Y.J., Kim C.D., Lee Y.H., Seo Y.J., Lee J.H., Lee Y. (2011). Can Platelet-rich Plasma Be Used for Skin Rejuvenation? Evaluation of Effects of Platelet-rich Plasma on Human Dermal Fibroblast. Ann. Dermatol..

[31] Correa-Gallegos D., Jiang D., Rinkevich Y. (2021). Fibroblasts as confederates of the immune system. Immunol. Rev..

[32] Palsson-McDermott E.M., Curtis A.M., Goel G., Lauterbach M.A., Sheedy F.J., Gleeson L.E., van den Bosch M.W., Quinn S.R., Domingo-Fernandez R., Johnston D.G. (2015). Pyruvate kinase M2 regulates Hif-1α activity and IL-1β induction and is a critical determinant of the warburg effect in LPS-activated macrophages. Cell Metab..

[33] Luo W., Hu H., Chang R., Zhong J., Knabel M., O’Meally R., Cole R.N., Pandey A., Semenza G.L. (2011). Pyruvate kinase M2 is a PHD3-stimulated coactivator for hypoxia-inducible factor 1. Cell.

[34] Zhou H.L., Zhang R., Anand P., Stomberski C.T., Qian Z., Hausladen A., Wang L., Rhee E.P., Parikh S.M., Karumanchi S.A. (2019). Metabolic reprogramming by the S-nitroso-CoA reductase system protects against kidney injury. Nature.

[35] Wong N., Ojo D., Yan J., Tang D. (2015). PKM2 contributes to cancer metabolism. Cancer Lett..

[36] Kang J.H., Jang Y.S., Lee H.J., Lee C.Y., Shin D.Y., Oh S.H. (2019). Inhibition of STAT3 signaling induces apoptosis and suppresses growth of lung cancer: Good and bad. Lab. Anim. Res..

[37] Bentov I., Damodarasamy M., Plymate S., Reed M.J. (2014). Decreased proliferative capacity of aged dermal fibroblasts in a three dimensional matrix is associated with reduced IGF1R expression and activation. Biogerontology.

[38] Li Y., Zhu L., Liu L., Li B. (2025). Role of macrophage PKM2 in inflammation and tumor progression and its targeted therapy. Biochim. Biophys. Acta Rev. Cancer.

[39] Wu Y.H., Cheng M.L., Ho H.Y., Chiu D.T., Wang T.C. (2009). Telomerase prevents accelerated senescence in glucose-6-phosphate dehydrogenase (G6PD)-deficient human fibroblasts. J. Biomed. Sci..

[40] Li J., Pang J., Liu Z., Ge X., Zhen Y., Jiang C.C., Liu Y., Huo Q., Sun Y., Liu H. (2021). Shikonin induces programmed death of fibroblast synovial cells in rheumatoid arthritis by inhibiting energy pathways. Sci. Rep..

[41] Herovici C. (1963). Picropolychrome: Histological staining technic intended for the study of normal and pathological connective tissue. Rev. Fr. Etud. Clin. Biol..

[42] Anthony P.P. (1975). Manual of Histological Demonstration Techniques. J. Clin. Pathol..

