# Supplementary material for: Leukocyte-Rich Platelet-Rich Plasma (L-PRP) Promotes Rejuvenation of Senescent Fibroblasts and Dermal Remodeling via CCL1-CCR8 Signaling and PKM2 Modulation
Source: Int J Mol Sci. 2026 Jul 15;27(14):6281. doi: 10.3390/ijms27146281 (PMC13410191; doi:10.3390/ijms27146281)
Supplement: Supplementary file 1 [file ijms-27-06281-s001.zip › ijms-4381777-supplementary.pdf]

International Journal of
Molecular Sciences

MDPI

Article

Leukocyte-Rich Platelet-Rich Plasma (L-PRP) Promotes Rejuvenation of Senescent Fibroblasts and Dermal Remodeling via CCL1-CCR8 Signaling and PKM2 Modulation

Seyeon Oh [1,2,†], Hyoung Moon Kim [1,3,4,†], Wook Oh [5], Gwahn Woo Cheon [1,4,6], Kyoungmi Lee [4,7], Kuk Hui Son [8,*] and Kyunghee Byun [1,4,9,*]

1 Functional Cellular Networks Laboratory, Lee Gil Ya Cancer and Diabetes Institute, Gachon University, Incheon 21999, Republic of Korea; md.mac12@gmail.com (H.M.K.)
2 LIBON Inc., Incheon 22006, Republic of Korea
3 Maylin Apgujeong Clinic, Seoul 06024, Republic of Korea
4 Department of Anatomy & Cell Biology, Gachon University College of Medicine, Incheon 21936, Republic of Korea
5 Maylin Clinic the Hyundai, Seoul 07335, Republic of Korea
6 Maylin Clinic, Ilsan, Goyang 10391, Republic of Korea
7 Regain Clinic, Incheon 22002, Republic of Korea
8 Department of Thoracic and Cardiovascular Surgery, Gachon University Gil Medical Center, College of Medicine, Gachon University, Incheon 21565, Republic of Korea
9 Department of Health Sciences and Technology, Gachon Advanced Institute for Health & Sciences and Technology (GAIHST), Gachon University, Incheon 21999, Republic of Korea
* Correspondence: dr632@gachon.ac.kr (K.H.S.); khbyun1@gachon.ac.kr (K.B.); Tel.: +82-32-460-3666 (K.H.S.); +82-32-899-6511 (K.B.)
† These authors contributed equally to this work.

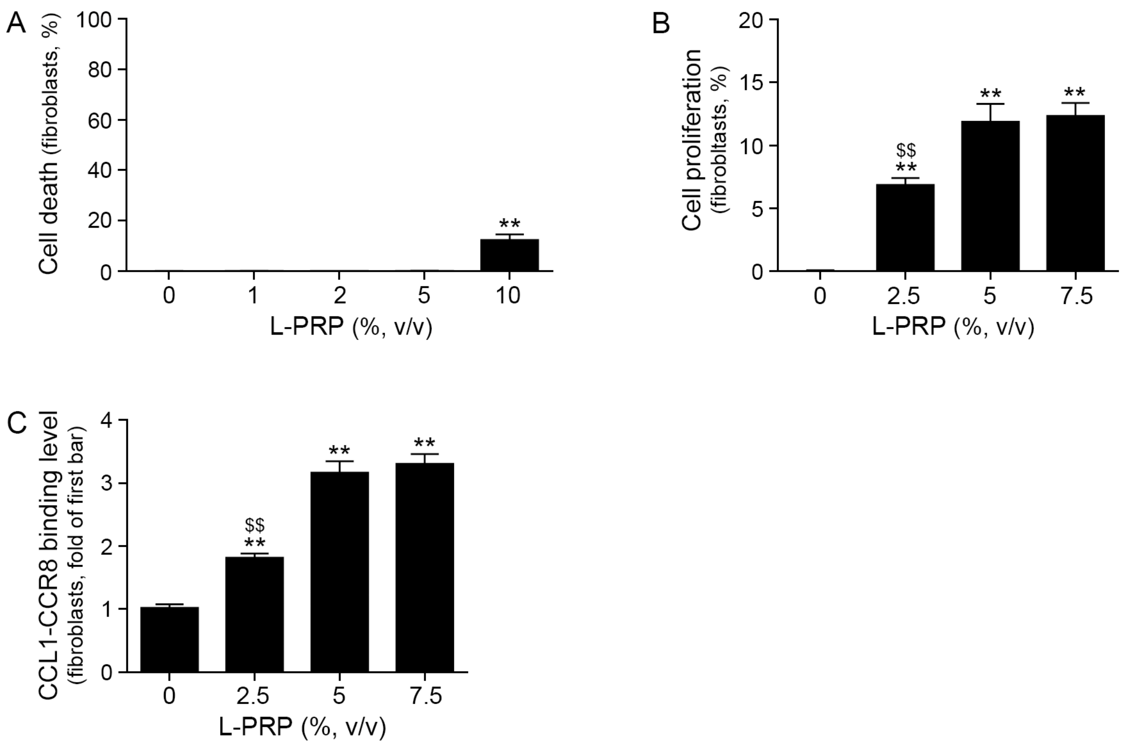

Figure S1. Assessment of the L-PRP concentration-dependent effects in H_2O_2-induced senescent HDFs. (**A**) Cell death (%) in H_2O_2 induced senescent HDFs treated with increasing concentrations of L-PRP (0–10% v/v) for 48 h, as determined by a CCK-8 assay. (**B**) Cell proliferation (%) in senescent HDFs treated with 0, 2.5, 5, or 7.5% (v/v) L-PRP for 48 h, demonstrating a dose dependent increase up to 5% L-PRP. (**C**) Relative CCL1-CCR8 binding levels (fold of untreated control) in senescent HDFs treated with the indicated concentrations of L-PRP, showing a plateau at 5% L-PRP. Data expressed as fold change relative to the first bar. Data are presented as the mean ± standard deviation (n = 5). Statistical significance was evaluated using the Kruskal–Wallis test followed by the Mann–Whitney U test; ***p* < 0.01, vs. first bar, $$*p* < 0.01, vs. third bar. CCK-8, cell counting kit-8; CCL, chemokine (C-C motif) ligand; CCR, C-C motif chemokine receptor; h, hour; HDFs, human dermal fibroblasts; H_2O_2, hydrogen peroxide; L-PRP, leukocyte-rich platelet-rich plasma.

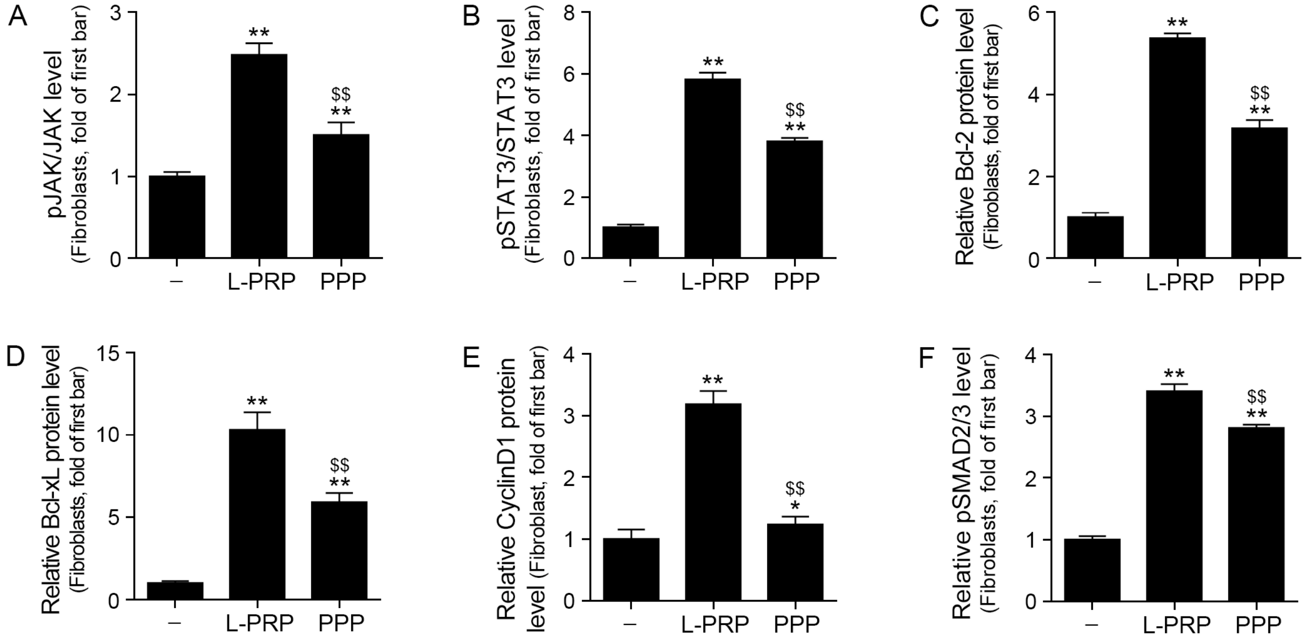

Figure S2. Densitometric quantification of western blot analyses related to JAK/STAT3 signaling and pSMAD2/3 activation in H_2O_2-induced senescent fibroblasts treated with L-PRP or PPP. (**A,B**) Quantification of pJAK/JAK and pSTAT3/STAT3 ratios in H_2O_2-induced senescent HDFs treated with 5% (v/v) L-PRP, or PPP. (**C–E**) Quantification of Bcl-2 (C), Bcl-xL (D), and Cyclin D1 (E) protein levels. (**F**) Quantification of nuclear pSMAD2/3 levels. Protein levels in panels A–E were normalized to β-actin, whereas nuclear pSMAD2/3 levels in panel F were normalized to histone H3. Values are expressed as fold change relative to the first bar. Data are shown as mean ± standard deviation (n = 5). Statistical analysis was performed using the Kruskal–Wallis test followed by the Mann–Whitney U test; *$p < 0.05$ and **$p < 0.01$, vs. first bar; $$$p < 0.01$, vs. second bar. HDFs, human dermal fibroblasts; H_2O_2, hydrogen peroxide; L-PRP, leukocyte-rich platelet-rich plasma; PPP, platelet-poor plasma.

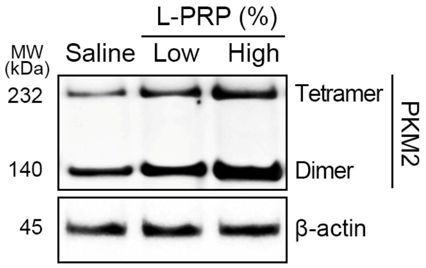

Figure S3. Full western blot images of PKM2 dimer and tetramer proteins in skin tissue. Full western blot images show PKM2 dimer and tetramer levels in skin tissues from saline- or L-PRP-injected mice. The corresponding cropped images are in Figure 3C and 4A. L-PRP, leukocyte-rich platelet-rich plasma.

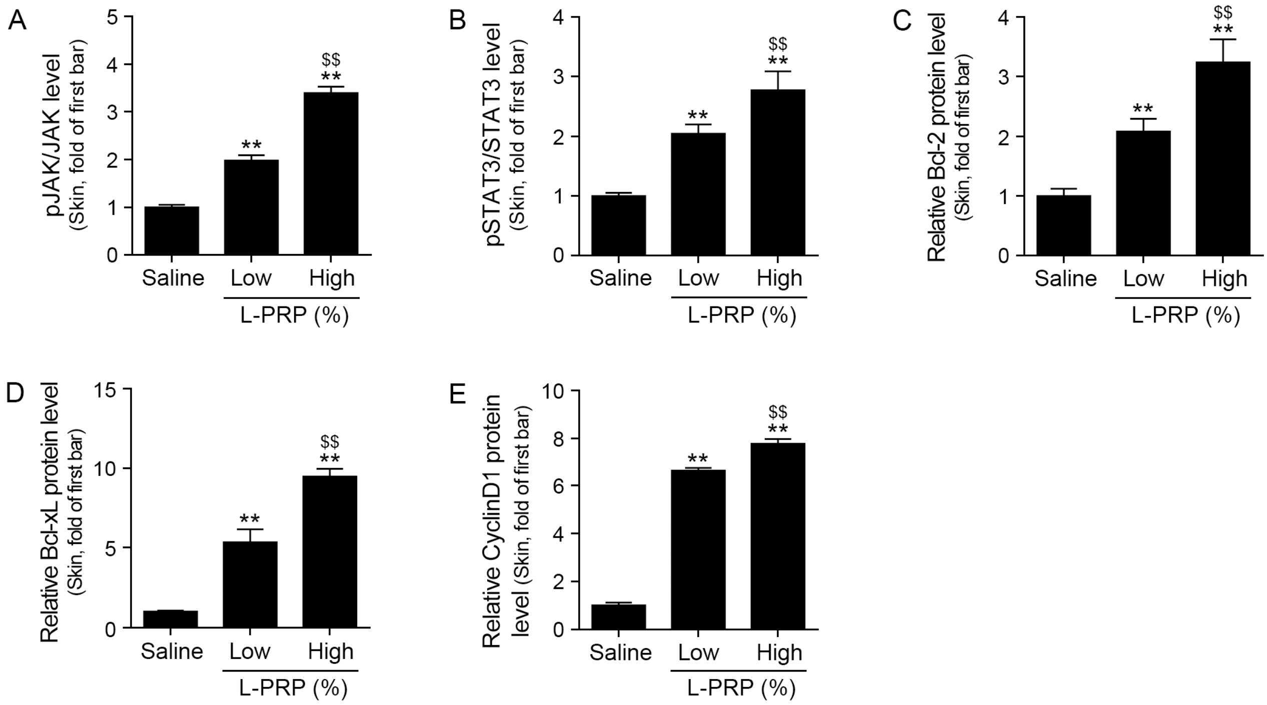

Figure S4. Densitometric quantification of western blot analyses related to JAK/STAT3 signaling in aged mouse skin injected with L-PRP. (**A,B**) Quantification of pJAK/JAK and pSTAT3/STAT3 ratios in skin tissues from saline- or L-PRP-injected 16-month-old mice. (**C–E**) Quantification of Bcl-2 (C), Bcl-xL (D), and Cyclin D1 (E) protein levels. Protein levels were normalized to β-actin and expressed as fold change relative to the first bar. Data are shown as mean ± standard deviation (n = 5). Statistical analysis was performed using the Kruskal–Wallis test followed by the Mann–Whitney U test; ***p* < 0.01, vs. first bar; $$*p* < 0.01, vs. second bar. L-PRP, leukocyte-rich platelet-rich plasma.

Table S1. List of antibodies used for ELISA, Western blot and immunohistochemistry.

Antibody	Dilution ratio		
	**ELISA**	**Western blot**	**Immunohistochemistry**
CCL1	1:5000		
CCR8	1:1000		
PKM2		1:1000	
pJAK		1:1000	
JAK		1:1000	
pSTAT3		1:1000	
STAT3		1:1000	
Bcl-2		1:500	
Bcl-xL		1:200	
CyclinD1		1:500	
pSMAD2/3		1:500	
β -actin		1:1,000	
H3		1:1,000	
PCNA			1:200
Collagen type I			1:100
Collagen type III			1:100
